# Food-Derived Extracellular Vesicles as Multi-Bioactive Complex and Their Versatile Health Effects

**DOI:** 10.3390/antiox12101862

**Published:** 2023-10-13

**Authors:** JuDong Yeo

**Affiliations:** Department of Food Science and Biotechnology of Animal Resources, Konkuk University, Seoul 05029, Republic of Korea; jyeo@konkuk.ac.kr

**Keywords:** exosome, nanoparticle, extracellular vesicle, ectosome, multi-vesicular body

## Abstract

Extracellular vesicles (EVs) are membrane-bound organelles that are generally released by eukaryotic cells and enclose various cellular metabolic information, such as RNA, meta-proteins, and versatile metabolites. The physiological properties and diverse functions of food-derived EVs have been extensively elucidated, along with a recent explosive upsurge in EV research. Therefore, a concise review of the health effects of food-derived EVs is necessary. This review summarizes the structural stability and uptake pathways of food-derived EVs to target cells and their health benefits, including antioxidant, anti-inflammatory, and anticarcinogenic effects, gut microbiome modulation, and intestinal barrier enhancement.

## 1. Introduction

Initially, studies on extracellular vesicles (EVs)—cell-derived particles surrounded by a lipid bilayer—focused on the clearance of waste or undesired substances from cells [1]. Recently, new insights have been shed regarding this naturally occurring liposome as a mediator of intracellular communication. EVs contain a high level of endosome-derived metabolites inside their membrane, and diverse molecules on the surface, such as proteins, enable them to bind to recipient cells [2]. Upon binding, their internal cargo is transferred to the recipient cells after being taken up through several mechanisms (i.e., endocytosis, phagocytosis, or membrane fusion). This process allows communication between cells at short and long distances by transferring significant information [2].

Sporadic observations of EVs have led to remarkable advances in related scientific fields in recent years [3]. All cellular organisms discharge EVs. In other words, EVs are secreted from the outer membrane by Gram-negative bacteria and there is a release of cytoplasmic membrane vesicles by Gram-positive bacteria, referred to as bacterial EVs [4]. EVs are also produced and released from eukaryotic cells in which they are involved in primary (pathological) physiological processes such as cellular homeostasis, cancer development, infection propagation, and cardiovascular diseases [5]. Moreover, lemon tissue, a plant-based food, secretes EVs containing various metabolites, such as RNAs, citrate, and vitamin C [6]. Thus, EVs are ubiquitous in natural sources such as animals, plants, and microorganisms.

**Figure 1:**
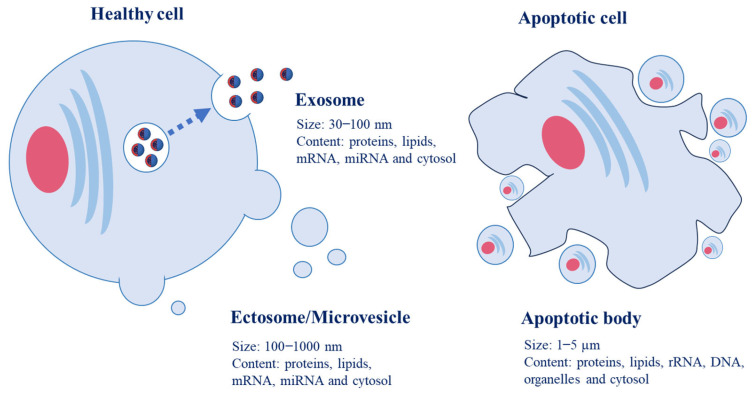
Pathway of development of extracellular vesicles such as exosomes, ectosomes/microvesicles and apoptotic bodies. Exosomes are small vesicles generated from intracellular endosomes. Ectosomes/microvesicles are membranous vesicles that are released via budding off from cell membranes. Apoptotic bodies are produced by blebbing or cell destruction during apoptosis.

EVs are classified into two types based on their biogenic processes: exosomes and ectosomes. Exosomes are endosomal-derived vesicles that are discharged upon the fusion of the plasma membrane and multivesicular bodies (MVBs) (Figure 1). They are unique endosomes encompassing intraluminal vesicles produced from invagination and budding of the limiting membrane, or amphisomes, that are hybrid organelles generated by the merging of autophagosomes and MVBs [7,8,9]. Ectosomes/microvesicles show a different pathway in the biogenesis process in that they are plasma membrane-derived EVs compared to exosomes. However, information on specific molecular markers for different biogenesis pathways remains insufficient, and only operational terms have been proposed to discriminate between EV types based on their biophysical or biochemical characteristics [2].

**Figure 2:**
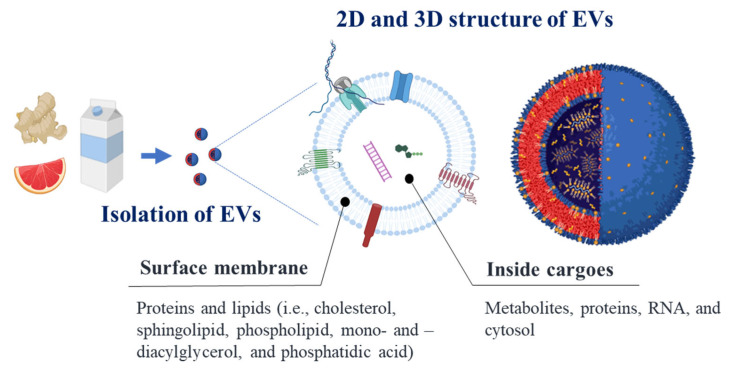
Structure of food-derived EVs and their molecular composition on the surface and internal cargoes.

Recent compelling evidence has demonstrated that most plant- and animal-based foods contain diverse types of EVs with different properties, such as size, cargo composition, origins, and surface molecules (Figure 2). For instance, exosome-like nanoparticles were isolated from coconut water, and the particle structures were observed using fluorescence staining after ultracentrifugation for isolation [10]. Ginger appears to yield a higher level of EVs compared to other plants, including grapes, carrots, and grapefruit; however, ginger-derived EVs seem to contain a higher RNA concentration than others [11]. Xiao et al. [12] obtained food-derived EVs from 11 different plant-based foods, including blueberry, coconut, ginger, grapefruit, Hami melon, kiwifruit, orange, pea, pear, soybean, and tomato, using a differential centrifugation approach. These EVs possess a large quantity of round or oval vesicles of different sizes (ranging from 100 to 1000 nm) and display a morphological structure similar to that of EVs from mammalian bodily fluids [13]. They also reported that these EVs are strongly associated with inflammatory and cancer-related pathways. Grapefruit-derived EVs have been demonstrated to possess lower RNA levels than other food-derived EVs and display a decrease in negative charge in the stomach, but not in the intestinal environment [11]. As explained above, various types of EVs have been found in animal- and plant-based foods, demonstrating their potential for multiple health effects.

Accumulating study has shown that animal cell-derived EVs exert excellent pharmacological activities by entering the body with a regular human diet and transferring miRNAs and nutrients. Despite the diverse health benefits of the EVs isolated from animal cells against many diseases (i.e., antioxidant, anti-inflammatory, and anticarcinogenic effects, gut microbiome modulation, and intestinal barrier enhancement), one of the significant disadvantages is the challenge in the production of the appropriate amounts of EVs in vitro and purifying those EVs from biological fluids. Indeed, the price and their clinical applications are primarily affected by the production efficiency of EVs per unit and are substantial [14]. EV production from cell cultures could also be risky due to the inherent harmful molecules inside EVs, such as substances having precancerous properties [15]. Conversely, plant-derived EVs are abundant in natural sources, enabling the isolation of large volumes and reducing production costs [16]: recent reports found high levels of EVs in grapes, tomatoes, and grapefruit by showing 1.76 mg/g, 0.44 mg/g, and 2.21 mg/g, respectively, indicating that certain plant-based foods could be utilized for the large-scale production of EVs [17]. Moreover, plant-derived EVs show high biocompatibility, low immunogenic nature, low toxicity, and a less allergic nature in the human body [18]. To date, research on the isolation and characterization of microbial-derived EVs is still insufficient compared to animal- and plant-derived EVs, and thus further research is needed to evaluate their potential value.

This review summarizes the physiological roles of EVs in biological systems (i.e., adaptive immunity, inflammation, allergic responses, and tumors) and their distribution in animal- and plant-based foods. Herein, we focused on the versatile health benefits of food-derived EVs as a novel type of multi-bioactive owing to their internal cargo. Therefore, this overview may be helpful for the development of food EV-based nutraceuticals, functional foods, and unique delivery systems.

## 2. Physiological Roles of EVs in Animal Cells

The diverse physiological roles of EVs in cellular space are now well demonstrated, and this section covers the various functions of EVs by focusing on immune responses, allergic responses, therapeutics, and diagnosis of cancer.

All immune cells participating in inflammation can discharge EVs; therefore, EVs have diverse functions in the inflammatory process. EVs exhibit a double-edged sword in sepsis by engaging in both pro-inflammatory and anti-inflammatory activities based on the types of donor cells and the phases of sepsis [19]. EV-associated cytokines have pro-inflammatory effects, whereas some EVs in sepsis show anti-inflammatory effects by downregulating acute-phase signaling and complement factors, suppressing leukocyte chemotaxis and decreasing serum pro-inflammatory cytokines [19].

EVs play significant roles in lymphocyte development; for instance, thymic epithelial cell-derived EVs are involved in the maturation of single-positive (CD4^+^ or CD8^+^) thymocytes by transporting proteins involved in thymic egress, such as sphingosine-1-phosphatase lyase 1 (SGPL1), dedicator of cytokinesis protein 2 (DOCK2), p21 protein-activated kinase 2 (PAK2), and Rho GDP-dissociation inhibitor 1 (GDIR1) [20]. EVs are also involved in B-cell development in immature primary bone marrow, in which they are responsible for the exchange of CD24 between B cells in the antibody-mediated engagement of CD24 [21].

EVs protect humans from microorganisms, such as Gram-positive and Gram-negative bacteria, fungi, and parasites [22]. Microbially derived EVs transport microorganism-associated molecular patterns, triggering host innate immune responses via pattern recognition receptors [23]. Interestingly, along with the EVs discharged by microorganisms, EVs released by infected cells also contain microbial molecules that may indirectly influence the immune response [24].

EVs are associated with allergic reactions and act as allergens and modulators. EVs in the plasma of patients with allergic rhinitis transport a high level of the house dust mite allergen Der p 1 compared to those in healthy controls, and plasma EVs from these patients cause a shift toward a T-helper 2 cell response [25]. Recent studies have demonstrated that interleukin 33, a cytokine that drives TH2 cell differentiation, is released by airway epithelial cells in association with the surface of exosomes [26]. Moreover, airway epithelial cell-derived EVs and EV-bound contactin 1 from individuals with asthma have been recognized as significant inducers of dendritic cell (DC) recruitment and stimulation of monocyte-derived DCs [27].

In cancer medicine, EVs have been presented as new targets that play a significant role in advancing cancer therapy and diagnosis. EVs influence tumor progression and metastasis by profoundly engaging in cell-to-cell communication [28]. Tumor-derived exosomes communicate with a broad spectrum of cells within the tumor microenvironment to induce tumor-friendly alterations that increase stromal activation, vascular permeability, immune escape of tumor cells, and the induction of an angiogenic switch [29]. Moreover, these tumor-derived exosomes support the establishment of a premetastatic niche, chemoresistance to neighboring cells, and the protection of tumor cells from the cytotoxic effects of immune cells and drugs [29]. Thus, the use of tumor-derived exosomes may contribute to advancing cancer diagnosis and treatment tools. In addition to the above roles, other physiological functions of EVs in animal cells have been documented in clinical research areas such as the central nervous system, neurodegenerative diseases, and brain homeostasis, as well as their broad applications in disease diagnosis and therapeutic perspectives [30].

## 3. Physiological Roles of EVs in Plant Cells

The essential and significant roles of EVs in the physiology and pathology of diverse plants have been well demonstrated. In plants, EVs are responsible for transporting genetic information across cellular spaces. Thus far, the transport of RNA through the rigid structure of plant cell walls is believed to be complex, and RNA outside cells may be too unstable to sustain their intact structure; however, findings on EVs in cellular space have provided new insights into the mobility of critical components, such as RNA, in the cellular metabolism of plants [31,32]. Notably, RNA mobility is deeply involved in morphogens and gene regulatory molecules that manage a variety of biological processes, including plant development, stress responses, nutrient allocation, root nodule symbiosis, and antiviral defense [31,32].

Regarding the mobility of EVs in cell wall matrices, it is known that the large size of EV particles restricts their capability of passing through the compact structure of cell wall matrices consisting of lignin, pectin, and cellulose fibers. However, recent studies have demonstrated passive diffusion by the dynamics of the cell wall, which could be at the locations of cell wall genesis or plant infection [33,34]. Moreover, the perturbation of the cell wall matrix by cell wall-degrading enzymes enables EV mobility [35,36,37].

Accumulating studies have proven that immune stress promotes EV secretion from plant cells; for instance, infection of Arabidopsis with *P. syringae pv tomato* DC3000 bacteria remarkably enhances EV release in extracellular fluids [35]. In addition, an increase in immune signaling in response to salicylic acid, a primary plant hormone required to maintain resistance against diverse pathogens, elevates the level of EVs in Arabidopsis extracellular fluids [35]. EVs function in the physiology and pathology of animal and plant cells, indicating their abundance in animal- and plant-based foods.

## 4. Structural Stability of Food-Derived EVs

The structural stability of food-derived EVs is a crucial factor in determining their functional maintenance and storage upon isolation and application to different systems. Various factors, such as isolation methods and sample types, greatly influence their stability [38,39]. Jang et al. [40] demonstrated the significance of isolation and purification methods. The combination of ultracentrifugation and the ExoQuick approach remarkably enhanced the colloidal stability of isolated ginseng exosomes by approximately double that of ultracentrifugation alone.

Furthermore, food-derived EVs isolated from different food sources show discrepancies in their stability during storage. Exosomes keep their structure for up to one year at −80 °C without coagulation after isolation from plant- and animal-based sources [2,41]. Munagala et al. [41] demonstrated that food-derived EVs had high stability at −80 °C for up to six months, while partial losses were observed at 4 °C. Exosomes have shown higher stability compared to other types of EVs, such as ectosomes and apoptotic bodies [42]. Exosomes have a liquid-ordered phase membrane, which leads to higher sensitivity to detergent lysis than other vesicle types [43]. Exosomes in biological fluids such as plasma sustain their intact structure for up to 5 days at 4 °C, while their stability is remarkably expanded upon storage at −20 °C for up to 3 months [44]. Human saliva exosomes maintain their structure after 28 days of storage at 4 °C in the presence of various enzymes [2]. Despite the high stability of animal cell-derived exosomes at low temperatures, their physicochemical characteristics are strongly influenced by other factors, such as repeated freeze–thaw cycles, pressure, and the nature of the solvent. The repetition of freeze–thaw cycles is a crucial factor in determining the shelf life and structural integrity of exosomes [42].

Food-derived EVs maintain their structure in an ex vivo digestion environment with only modest alterations in size and charge [45,46]. Furthermore, some orally administered food-derived EVs survive in the digestive tract and are introduced into the large intestine, where they suppress gut inflammation or approach liver cells [47]. Similarly, López de Las Hazas et al. [48] reported that miRNAs protected by bovine milk-derived exosomes showed higher stability than free miRNAs, as well as enhanced digestive tolerance, retarding the degradation rate of certain miRNAs in mice. However, food-derived EVs sustain these functions only when the membrane is intact. Therefore, care must be taken to ensure that their structures are not destroyed during storage and processing [49]. To date, the stability of food-derived EVs during storage and food processing has not been extensively investigated. Additional studies are needed to fill the existing gaps in related knowledge.

## 5. Absorption and Uptake of Food-Derived EVs

Orally administered food-derived EVs (i.e., ginger, grape, grapefruit, and carrot) appear to be absorbed via intestinal stem cells and macrophages at a similar rate in a confocal analysis of mouse intestinal tissues [12]. Although nonspecific uptake is common in all cell types, the uptake of EVs via specific targeting to recipient cells is the best pathway for transfer inside the cargo to exert their functions [50,51]. This process is managed by the molecular composition of the surface of EVs; for example, rabies viral glycoprotein on the surface of EVs, which specifically binds to the acetylcholine receptor, and enables the introduction of EVs into the brain [52,53,54]. The maintenance of tropism between cells is another mechanism of exosome-targeting specificity, in which the cellular signature preserved in the released exosomes functions as a recognition motif for absorption by the same types of recipient cells, as proven in in vitro and in vivo models [55,56].

Various studies have documented the uptake of food-derived EVs in vitro and in vivo. Zhang et al. [46] reported that gut macrophages and intestinal stem cells are responsible for the absorption of ginger-derived EVs in the colon. Interestingly, when orally administered to mice, ginger-derived EVs displayed greater retention in the colon in the starved mouse model than in the non-starved model, indicating the influence of the food digestion process (or digested food matrices) on the retention time of ginger-derived EVs in the colon.

In in vivo and in vitro tests, orally administered grape-derived EVs passed through the mucus barrier of the mouse intestine, followed by transportation to intestinal stem cells, which encouraged the proliferation of Lgr5+ stem cells through the catenin pathway [49]. Moreover, Syrah grape-derived EVs can pass through the intestinal tract, leading to the proliferation of Lgr5+ stem cells [45]. Rani et al. [57] also reported that miRNAs encapsulated in milk-derived exosomes not only showed high resistance against various digestive fluids (i.e., saliva, bile, gastric juice, and pancreatic juice) but also passed through the intestinal barrier via trans-epithelial transit to enter the blood circulation. Wolf et al. [58] investigated the transport mechanism of bovine milk-derived exosomes using fluorophore-labeled bovine milk-derived exosomes in rat small-intestine IEC-6 cells and human colon carcinoma Caco-2 cells. These results demonstrate that in rat and human intestinal cells, the absorption of bovine milk-derived exosomes is facilitated via endocytosis and influenced by glycoproteins on the surface of exosomes. They also reported the absorption of bovine milk-derived exosomes by human macrophages, but did not elucidate the detailed transport mechanism [59].

Upon reaching recipient cells, the cargo inside the EVs can be released via direct interaction, fusion with the plasma membrane, or internalization. In the direct interaction pathway, transmembrane ligands on the surface of EVs interact directly with the receptors of the recipient cell and immediately produce a downstream signaling cascade to stimulate the target cell [60]. Food-derived EVs may also release their internal cargo into the cytosol of recipient cells via fusion with the plasma membrane. Moreover, EVs can transport and discharge their internal components via internalization pathways (clathrin-mediated endocytosis, caveolin-mediated endocytosis, lipid raft-mediated endocytosis, micropinocytosis, or phagocytosis). Thus, food-derived EVs may reach the target cells, followed by their uptake through the pathways described above [61].

## 6. Health Benefits of Food-Derived EVs

**Figure 3:**
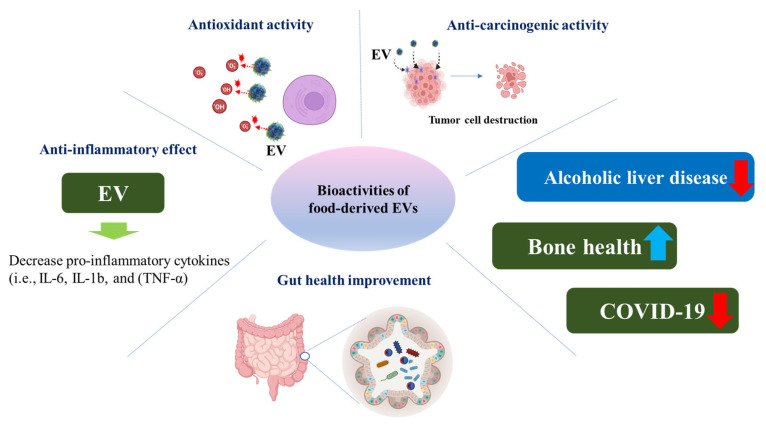
Various health benefits of food-derived EVs.

In this section, various health benefits of food-derived EVs are discussed by focusing on antioxidant activity, anti-inflammatory effect, anticarcinogenic activity, gut microbiome modulation and intestinal barrier enhancement (Figure 3).

### 6.1. Antioxidant Activity

Reactive oxygen species (ROSs) are produced during aerobic respiration and in response to cytokines, xenobiotics, and bacterial invasion, and are involved in cell survival and proliferation [62]. An imbalance between the antioxidant defense and free radical concentration leads to oxidative stress in cells. Notably, the overproduction of ROS may result in oxidative damage to essential cell components, including DNA, proteins, and lipids, further affecting the etiology of diverse diseases, such as heart diseases, neurodegenerative disorders, and diabetes [63]. Evidence has been reported on the antioxidant potential of plant-derived EVs as novel antioxidant complexes.

De Robertis et al. [64] demonstrated the uptake of blueberry-derived EVs in a human endothelial cell model in a dose-dependent manner. The absorbed EVs constrained the generation of ROSs, followed by enhanced cell viability. Perut et al. [65] isolated plant-derived exosome-like nanovesicles (EPDENs) from strawberry juice containing high levels of anthocyanins, folic acid, flavonols, and vitamin C. They demonstrated their strong antioxidant capacity by suppressing oxidative stress in human mesenchymal stromal cells (MSCs) in a dose-dependent manner. Grapefruit and tomato exosome-like vesicles display weak antioxidant potential compared to their juice sources in vitro tests, such as radical scavenging capacities and cell viability assays [66]. Aloe vera peel-derived EVs exhibited high antioxidant potential in superoxide dismutase (SOD) activity and cellular antioxidant activity assays: they remarkably reduced the level of intracellular ROSs in a dose-dependent manner in H_2_O_2_-treated HaCaT cells [67]. Moreover, aloe vera peel-derived EVs upregulated the mRNA expression of Nrf2, CAT, HO-1, and SOD.

Exosomes isolated from citrus fruits and berries are highly stable in the gastric tract before entering the small intestine [68]. They possess a high level of vitamin C as their cargo; for instance, there is 7 µM vitamin C in 50 µg/mL of lemon exosomes, leading to their potent antioxidant ability by effectively delivering high doses of antioxidants to the target cells [69]. Lemon-derived EVs are one of the most investigated citrus-based exosomes, which were isolated using a centrifugation approach from lemons (*Citrus limon* L.). They showed antioxidant activity in an MSC model due to the RNAs, citrate, and vitamin C preserved inside the lemon-derived EVs. Lemon-derived EVs also significantly enhance the survival of mesenchymal stem cells in H_2_O_2_-induced oxidation in a concentration-dependent manner by suppressing ROS production [6]. Carrot-derived EVs significantly alleviated ROS production in Parkinson’s disease (PD) and myocardial infarction models by effectively suppressing the expression of antioxidant molecules (i.e., Nrf-2, nuclear factor erythroid 2-related factor 2, NQO-1, and HO-1) [70]. A similar study showed the antioxidant properties of carrot-derived EVs. They elevated the nuclear translocation of Nrf2, which is a primary regulator of the heme oxygenase-1 (HO-1) gene engaged in antioxidative activity, in a RAW 264.7 macrophage model [12]. Increasing evidence has demonstrated that 6-shogaol stimulates the expression of Nrf2, followed by its contribution to hepatoprotection [13].

Blueberry-derived EVs attenuated oxidative stress in mouse models fed a high-fat diet, in which EVs alleviated ROS concentration by interacting with Bcl-2 mitochondrial protein functionality, which inhibited cell apoptosis in HepG2 cells [71]. Tea leaf-derived EVs also suppress the production of ROS in RAW 264.7 macrophages due to the high level of galactose-functionalized proteins on the surface of EVs [72].

### 6.2. Anti-Inflammatory Effect

Inflammation is an essential bioprocess that responds to external stimuli, including injury, infection, and irritation, by secreting pro-inflammatory cytokines [73]. However, despite their crucial roles, the overproduction of pro-inflammatory cytokines, i.e., IL-6, IL-1b, and tumor necrosis factor alpha (TNF-α), induces severe adult diseases, such as allergy, arthritis, atherosclerosis, and cancer [74]. Therefore, suppressing the overproduction of pro-inflammatory cytokines is critical for inhibiting the occurrence of relevant ailments.

Teng et al. [47] demonstrated that ginger-derived EVs are taken up by certain bacterial species that reside in the intestinal tract of mice and that the miRNAs inside EVs contribute to anti-inflammatory effects by influencing microbial composition. Notably, mdo-miR-7267-3p enhanced the regulation of the mRNA expression of the ycNE in the microbiome, leading to an improvement in the production of indole-3-carboxaldehyde (I3A), which in turn affected the growth of the gut microbiota and promoted the production of IL-22, a cytokine closely associated with a decrease in the inflammatory response. Moreover, ginger-derived EVs induced inflammatory responses by inhibiting the regulation of the expression of NF-κB, IL-8, TNF-α, and IL-6 in Caco-2 cells [75]. Arntz et al. [76] demonstrated that bovine milk-derived EVs suppressed miR-30-a, miR-92-a, miR-223, β-lactoglobulin mRNA, and β-casein mRNA, which may be absorbed by RAW 264.7, intestinal cells, and splenocytes, contributing to the retardation of arthritis in murine models.

Ginger-derived EVs elevated the gene expression of both anti-inflammatory cytokines (i.e., HO-1 and IL-10) and pro-inflammatory cytokines such as IL-6 and TNFα at a much higher rate compared to other food-derived EVs, showing effectiveness in maintaining gut homeostasis [12]. Ginger-derived EVs also suppress the levels of lipocalin-2, a biomarker of gut inflammation, and do not affect cell viability or cause any side effects [12]. Grapefruit-derived EVs exert anti-inflammatory effects upon absorption by intestinal macrophages by inhibiting the expression of pro-inflammatory cytokines, including IL-6 and TNF-α [77]. The authors concluded that this effect might be due to naringin and naringenin inside the grapefruit-derived EVs; however, they argued that further research is required to prove the contribution of these bioactive compounds to the anti-inflammatory effect [77]. Shiitake mushroom-derived EVs inhibited the formation of macrophage NLRP3 inflammasome and pro-inflammatory cytokines such as IL-1β, while six other mushroom-derived EVs tested did not inhibit the generation of NLRP3, showing discrepancies in anti-inflammatory effects of EVs depending on the mushroom species [78]. Exosomes obtained from macrophage cell lines and murine lymphomas have been used to deliver curcumin: exosome encapsulation significantly improves the anti-inflammatory capacity of curcumin and increases target specificity toward inflammatory cells [79]. Therefore, food-derived EVs are involved in inflammatory reactions via diverse pathways.

Exosomes themselves play a key role as mediators of the inflammatory response in biological systems; therefore, immune cells secrete exosomes along with changed cargo upon recognizing external stimuli [80]. For instance, RAW 264.7 macrophages discharged elevated levels of exosomes containing an increased concentration of proteins upon stimulation with lipopolysaccharide endotoxins, which induces the secretion of IL-6 and TNF-α in macrophages [81]. Moreover, DCs secrete exosomes, leading to alterations in gene expression in recipient T cells. T cells also respond to foreign stimuli by secreting exosomes that activate resting T cells [82,83].

### 6.3. Anticarcinogenic Activity

Anticarcinogenic activity is known to retard the transformation of normal cells, angiogenesis, tumor growth, and metastasis. Food-derived EVs can modify gene expression in cancer cells upon uptake, suppressing cancer-related phenotypes. Recent studies have demonstrated the anticarcinogenic activity of food-derived EVs isolated from a broad spectrum of natural sources.

Plant-derived EVs are promising novel anticarcinogenic substances and are considered promising alternatives for current antitumor treatments; for instance, miRNAs secreted from food-derived EVs are responsible for the proliferation and apoptosis of tumors [84,85]. Bovine, porcine, and human breast milk-derived EVs possess miR-148a, which leads to antitumor activities by regulating the expression of genes closely associated with tumor development and proliferation, including DNMT1, ROCK1, and ERBB3 [86,87]. Samuel et al. [88] documented that milk-derived EVs rich in miRNAs constrain tumor growth; however, they may accelerate cancer metastasis in pancreatic and breast cancer mouse models. Ginger-derived EVs containing 125 miRNAs have been shown to reduce pro-inflammatory cytokine levels in mouse colitis models [46]. Furthermore, this study provides detailed insights into EV miRNA payloads in the cross-species regulation of cancers.

Zhang et al. [46] isolated EVs from ginger and converted them into novel nanoparticles by reassembling their composition. The reassembled nanoparticles efficiently enclosed doxorubicin (Dox) inside their structure, transferring it into the tumor and inhibiting its growth. EVs from lemon suppress the proliferation of three types of cancer cells—A549, SW480, and LAMA84—at a concentration of 20 μg/mL lemon-derived EVs by promoting the expression of pro-apoptotic genes of tumor cells [89]. The anticancer activity of lemon-derived EVs has also been demonstrated in vivo, where the introduction of EVs into the tumor sites of LAMA84-inoculated mice by both intraperitoneal and local injections significantly inhibited tumor growth and size [89]. Additionally, Karlsson et al. [90] reported that lemon-derived EVs improve TNF-related apoptosis, such as ligand (TRAIL)-mediated apoptosis, which is responsible for the decrease in angiogenic cytokine secretion (e.g., vascular endothelial growth factor-α, IL-8, and IL-6). These EVs also target acetyl-CoA carboxylase α and phosphatidic acid-preferring phospholipase A1, indicating a targetable deregulation in cancer therapies. Moreover, grape-derived EVs alleviate oral mucositis during chemoradiation in neck and head cancers (NCT01668849) [91]. Grape-derived EVs have also been suggested as novel solutions for transporting anticancer agents by addressing the limitations of synthetic liposomes in terms of bioavailability, safety, and stability [91].

### 6.4. Gut Microbiome Modulation and Intestinal Barrier Enhancement

The composition of the gut microbiota is greatly affected by dietary interventions and is involved in the occurrence of several diseases [92]. Teng et al. [47] reported that plant-derived EVs could be taken up by the gut microbiome, causing alterations in gut microbiota profiles and host physiology due to internal components such as RNAs. For instance, the uptake of ginger-derived EVs enclosing certain microRNAs preferentially occurs in *Lactobacillaceae* and induces the targeting of specific genes in *Lactobacillus rhamnosus*. This complicated gene-targeting mechanism increases the expression of indole-3-carboxaldehyde (I3A), followed by the induction of IL-22 production. In summary, the above evidence demonstrates that the uptake of food-derived EVs by the gut microbiota may improve intestinal barrier function and inhibit colitis in mouse models via an IL-22-dependent mechanism [47].

The introduction of food-derived EVs is closely related to the composition of the gut microbiota. Plant- and milk-derived EVs containing miRNAs can be absorbed by bacteria and actively regulate the expression of specific genes that affect microbial growth [92,93]. The miRNAs present in milk-derived exosomes influence gut microbiota profiles; in particular, they enhance the growth of certain bacteria, including *Firmicutes*, *Lachnospiraceae*, and *Tenericutes* [94]. Moreover, Tartary buckwheat-derived EVs improve the diversity of the intestinal microbiota and stimulate target functional genes that affect the physiological processes of *Lactobacillus rhamnosus* and *Escherichia coli* and their growth as well [95].

Meanwhile, intestinal epithelial cells function as a barrier and play a crucial role in preventing and controlling the entry of antigens and pathogenic toxins into the systemic circulation, as well as allowing the translocation of luminal nutrients, water, and electrolytes released from the intestinal microbiota and intestinal tissue [96]. Several reports have documented improvements in intestinal barrier function by food-derived EVs and their cargo. miRNAs present in plant- and milk-derived EVs can penetrate the gastrointestinal tract and play critical roles in enhancing the permeability and integrity of the intestinal barrier, where they engage in multiple pathways, such as intestinal epithelial cell modification, gut microbiota regulation, and intestinal immune system improvement [97,98,99]. In addition, orally administered fruit-derived EVs promote the signaling process of Wnt/β-catenin in stem cells of the intestinal barrier, leading to improved cell proliferation, which enhances the homeostasis and integrity of the intestinal wall [12,49]. Zhang et al. [46] documented that orally administered ginger-derived EVs induced cell proliferation and promoted the expression of adherent junction proteins (i.e., E-cadherin and desmoglein) by internalizing the consumed EVs in the colon epithelial cells of mice with colitis. Moreover, broccoli-derived EVs inhibited colitis by regulating intestinal immune homeostasis by targeting DCs in a mouse colitis model [100].

Based on the large dataset reported, it is assumed that food-derived EVs may shed new light on regulating intestinal barrier permeability and the intestinal immune system and modulating gut microbiota composition.

### 6.5. Inhibition of the Effect of COVID-19

EVs isolated from dietary sources have an impact on suppressing the effect of COVID-19, a virus that—owing to high levels of small RNA—has caused widespread death in the absence of adequate treatment and the largest global economic crisis [101]. EVs discharged by severe acute respiratory syndrome coronavirus type 2 (SARS-CoV-2) cells seem to cause pulmonary inflammation, in which ginger-derived EVs reduce Nsp12 gene expression and inhibit the damage caused by SARS-CoV-2 in the lungs [102], indicating that the small RNA enclosed in food-derived EVs could be a potential treatment for COVID-19.

### 6.6. Suppression of Alcoholic Liver Disease

Food-derived EVs inhibit alcoholic liver disease. Ginger-derived EVs appeared to shield liver damage from alcohol-induced stimulation in mouse models by activating NRF2 and enhancing the expression of specific genes relevant to liver detoxification and antioxidant activity, while reducing the generation of ROSs [13]. Moreover, *Lentinus edodes*-derived EVs protect mice from liver injury induced by D-galactosamine/lipopolysaccharide by restraining the activation of NLRP3 [82]. Accordingly, food-derived EVs can be used to treat alcoholic liver disease.

### 6.7. Improvement in the Growth of Probiotics

Some studies have demonstrated the effect of food-derived EVs on the growth of several probiotics, whereas others have shown a suppression of the growth of harmful bacteria. Increasing evidence has shown that some food-derived EVs can be absorbed by bacteria upon co-incubation in appropriate environments [103]. miRNAs inside EVs may play a crucial role in controlling bacterial growth, i.e., promoting the growth of probiotics owing to miRNAs in coconut water-derived EVs [101]. EVs isolated from *Arabidopsis thaliana* can transfer small RNAs to the site of fungal infection, thereby supporting the downregulation of fungal genes [101].

### 6.8. Enhancement in Immune Systems

Food-derived EVs play crucial roles in developing and maintaining the immune system by delivering immune-related miRNA and proteins, which support immunomodulatory functions. For instance, Matic et al. [104] demonstrated that bovine milk-derived exosomes improved the proliferation of RAW 264.7 cells, suppressed cisplatin-induced cytotoxicity, and controlled the production of proteins associated with the cell cycle. Moreover, Ascanius et al. [105] reported that milk-derived EVs decreased the expression of inflammatory cytokines and reduced NK-κB activity in LPS-treated RAW 264.7 cells. However, cow milk-derived EVs did not improve the functions of immune cells [106].

## 7. Other Bioactivities of Food-Derived EVs

In addition to the bioactivities described above, many studies have demonstrated other health benefits of food-derived EVs. A recent study documented that *Porphyromonas gingivalis*, a gum disease pathogen, appears to selectively uptake ginger-derived EVs via the interaction between phosphatidic acid on the EVs’ membrane and hemin-binding protein 35 (HBP35) on the exterior of *P*. *gingivalis* [103]. Upon uptake of EVs, the cargoes present in EVs are released into the internal bacteria, which leads to a decrease in their pathogenicity and potential to attack oral epithelial cells, followed by the prevention of bone loss of the teeth caused by *P. gingivalis* [103]. Lemon-derived EVs suppress the mortality of mice infected with *Clostridioides difficile*. These EVs enhance the viability of probiotics, such as *Lactobacillus rhamnosus* GG and *Streptococcus thermophilus* ST-21, resulting in reduced mortality of the infected mice by increasing the secretion of lactic acid in the gut to hinder the growth of *C. difficile* [107].

Luo et al. [108] demonstrated the neuroprotective activity of bovine milk-derived exosomes containing epicatechin gallate (ECG) along with their average size of 85.15 ± 2.00 nm against a rotenone (Rot)-induced PD model. This showed that ECG was successfully transported into SHSY5Y cells following their neuroprotective effects. Moreover, the immunoregulatory actions of milk-derived EVs against the infant immune system have been explained by the presence of miRNAs in EVs. Xie et al. [109] demonstrated that porcine milk-derived EVs containing miRNAs confer high stability against LPS-induced intestinal inflammation and apoptosis to intestinal epithelial cells. In this process, miR-4334 and miR-219 inside EVs suppressed intestinal inflammation by regulating the TLR4/NF-κB pathway, while miR-338 repressed apoptosis through p53 pathway regulation and handled severe damage in intestinal epithelial cells in a TLR4-dependent mechanism [110].

The health benefits of food-derived EVs extend to their potential as new platforms for nutraceuticals and functional foods. Citrus lemon-derived EVs significantly enhance collagen synthesis, improve the maintenance of bone matrix structure, and manage bone health [6]. Food-derived EVs also display wound healing potential: aloe vera-derived EVs increase the mobility of fibroblasts and keratinocytes to the wound site, proving their potential as a promising source for skin regeneration therapy [70]. EVs isolated from wheatgrass juice increase the proliferation and migration of endothelial cells (HUVEC), dermal fibroblasts (HDF), and epithelial cells (HaCaT), supporting the fact that food-derived EVs could be an effective natural source for developing wound healing and cosmetic products [111].

## 8. Conclusions and Perspectives

Much evidence has been documented regarding the effects of food-derived EVs on physiological events and their potential applications in the treatment of human diseases, offering new insights into therapeutic solutions. Promising results from the application of food-derived EVs, directly or with manipulation, to different diseases such as cancer, inflammation, and gastrointestinal pathologies suggest a new platform for natural source-based bioactive compounds. Despite the above findings of extant research, additional efforts should be made to fill the existing gaps in understanding the properties of food-derived EVs, such as their stability in the digestive tract and their alteration upon food processing and storage, before their extensive application in related fields. Moreover, further studies should focus on understanding the detailed mechanisms behind their biogenesis, kinetics, and trafficking, as well as their broad applications to therapeutic effects and promising potential as a novel delivery system.
